# Post-COVID-19 recovery and geriatric rehabilitation care: a European inter-country comparative study

**DOI:** 10.1007/s41999-024-01030-w

**Published:** 2024-08-13

**Authors:** Lisa S. van Tol, Tiangao Lin, Monique A. A. Caljouw, Matteo Cesari, Frances Dockery, Irma H. J. Everink, Bahaa N. Francis, Adam L. Gordon, Stefan Grund, Luba Matchekhina, L. Mónica Perez Bazan, Eva Topinková, Mark A. Vassallo, Wilco P. Achterberg, Miriam L. Haaksma, on behalf of the EU-COGER consortium

**Affiliations:** 1 Department of Public Health and Primary Care, Leiden University Medical Center, Leiden, The Netherlands; 2 Center for Medicine for Older People, Leiden University Medical Center, Leiden, The Netherlands; 3 University Network for the Care Sector South-Holland, Leiden University Medical Center, Leiden, The Netherlands; 4 Department of Physical Medicine and Rehabilitation, Sir Run Run Shaw Hospital, Hangzhou, China; 5 IRCCS Istituti Clinici Maugeri, University of Milan, Milan, Italy; 6 Beaumont Hospital & Royal College of Surgeons in Ireland, Dublin, Ireland; 7 Department of Health Services Research, Maastricht University, Maastricht, The Netherlands; 8 Fliman Geriatric Rehabilitation Center, Haifa, Israel; 9 Geriatric Division, Holy Family Hospital, Bar Ilan University, Safad, Israel; 10 Academic Unit of Injury, Recovery and Inflammation Sciences (IRIS), School of Medicine, University of Nottingham, Nottingham, UK; 11 NIHR Applied Research Collaboration-East Midlands (ARC-EM), Nottingham, UK; 12 Center for Geriatric Medicine, Agaplesion Bethanien Hospital Heidelberg, Geriatric Center at the Heidelberg University, Heidelberg, Germany; 13 Russian Gerontology Research and Clinical Centre, Pirogov Russian National Research Medical University, Moscow, Russia; 14 RE-FiT Barcelona Research Group, Parc Sanitari Pere Virgili Hospital and Vall d’Hebron Institut de Recerca (VHIR), Barcelona, Spain; 15 Department of Geriatrics, First Faculty of Medicine, Charles University and General Faculty Hospital, Prague, Czech Republic; 16 Faculty of Health and Social Sciences, University of South Bohemia, Ceske Budejovice, Czech Republic; 17 Karin Grech Hospital, Pieta, Malta

**Keywords:** Geriatric rehabilitation, COVID-19, Recovery, Europe

## Abstract

**Purpose:**

There is variation in organization of geriatric rehabilitation across Europe. The purpose of this study was to describe the selection criteria for referral to geriatric rehabilitation, care provided, and recovery trajectories of post-COVID-19 patients referred to geriatric rehabilitation in Europe.

**Methods:**

This observational cohort study included 723 patients in 59 care facilities for geriatric rehabilitation across 10 countries. Patient data were collected from medical records on admission to geriatric rehabilitation (between September 2020 and October 2021), discharge, 6 weeks and 6 months follow-up. The primary and secondary outcomes were recovery in daily functioning (Barthel Index) and Quality of Life (EQ-5D-5L) from admission to discharge. These were examined using linear mixed models with two levels (measurements nested in patients) and country as an independent variable. Random intercept and random linear slope parameters were added when they improved model fit. A survey about organization of geriatric rehabilitation for post-COVID-19 patients was filled out by country coordinators and data were analyzed using descriptive statistics and inductive coding of answers to open questions.

**Results:**

Patients had a mean age of 75.7 years old and 52.4% were male. Many countries used various combinations of the selection criteria, such as functional status, age, frailty, Comprehensive Geriatric Assessment, comorbidities, and cognitive impairments. Most patients received physiotherapy (88.8%) and occupational therapy (69.7%), but there was substantial variance between countries in the percentages of patients that received protein or calorie enriched diets, oxygen therapy, and other treatment components. In all countries, patients showed recovery in daily functioning and quality of life, although there was variation in between countries in rate of recovery. Daily functioning seemed to increase most rapidly in the Czech Republic, Germany, and Russia. The steepest increases in quality of life were seen in the Czech Republic, Germany, and Spain.

**Conclusion:**

Post-COVID-19 patients showed recovery during geriatric rehabilitation, albeit at variable rates. The observed variation may be explained by the heterogeneity in selection criteria and care provided. This study highlights the need for harmonization of measurements in geriatric rehabilitation order to perform explanatory research and optimize geriatric rehabilitation throughout Europe to ensure optimal patient recovery.

**Supplementary Information:**

The online version contains supplementary material available at 10.1007/s41999-024-01030-w.

## Key summary points

**Aim:**

To describe selection criteria for referral to geriatric rehabilitation, care provided, and recovery trajectories of post-COVID-19 patients referred to geriatric rehabilitation in Europe.

**Findings:**

In the ten participating countries, patients showed recovery in daily functioning and quality of life, albeit at variable rates. This variation in recovery rates was accompanied by variation in geriatric rehabilitation selection criteria, patient characteristics, and provided rehabilitation care.

**Message:**

The heterogeneity in recovery of post-COVID-19 patients admitted to geriatric rehabilitation, selection criteria, and organization of geriatric rehabilitation care highlights the need for harmonization of measurements in geriatric rehabilitation in order to perform explanatory research and optimize geriatric rehabilitation throughout Europe.

**Supplementary Information:**

The online version contains supplementary material available at 10.1007/s41999-024-01030-w.

## Introduction

Millions of people have been infected with Severe Acute Respiratory Syndrome Coronavirus 2 (SARS-COV-2) since the start of the COVID-19 pandemic in 2020 [1]. Although COVID-19 is no longer called a public health emergency since May 2023, the end of the pandemic is not yet in sight [2]. Infections and deaths still occur [1] and new virus variants may again cause increased infection rates and disease outbreaks. Older age is strongly associated with increased risk of severe COVID-19 infection and death [3–5].

The large number of older patients with COVID-19 has led to increased demand for geriatric rehabilitation. The European Geriatric Medicine Society (EuGMS) has defined geriatric rehabilitation as “a multidimensional approach of diagnostic and therapeutic interventions, the purpose of which is to optimize functional capacity, promote activity and preserve functional reserve and social participation in older people with disabling impairments” [6]. Unlike rehabilitation for specific diseases, geriatric rehabilitation is tailored to specific needs and appropriate goals for older people who more often experience multiple long-term conditions and geriatric syndromes such as frailty [7]. In many European countries geriatric rehabilitation is still underdeveloped [8]. A survey by the EuGMS revealed that, in 2018, geriatric rehabilitation was only recognized formally in two-thirds (20 out of 31) of participating European countries, and national or local geriatric rehabilitation guidelines were in use in only one-third (11 out of 31) [8].

The COVID-19 pandemic increased demand for geriatric rehabilitation, but also reduced its capacity. This has been called the COVID “rehabilitation paradox” [9]. Reasons for reduced capacity included pandemic-related spacing requirements, adapted admission criteria, and GR beds being repurposed to deliver acute care [9]. Moreover, GR facilities had staff shortages due to illness or secondment to acute care wards.

Despite efforts of expert groups to provide guidance on geriatric rehabilitation in post-COVID patients [10], scientific evidence on how best to organize care to facilitate recovery for geriatric patients after COVID-19 is limited. More insight into geriatric rehabilitation care provided during the pandemic in various European countries and the recovery of geriatric patients after COVID-19 are needed for countries to be able to learn from each other, optimize COVID-19 rehabilitation and prepare for potential future pandemics. This study aims to describe selection criteria for referral to geriatric rehabilitation, care provided, and recovery of patients after COVID-19 in geriatric rehabilitation across multiple European countries.

## Methods

### Design

This study was a part of the European Cooperation in Geriatric Rehabilitation study after COVID-19 (EU-COGER). EU-COGER was an international observational cohort study designed by the EuGMS Special Interest Group for Geriatric Rehabilitation. The study was registered at ClinicalTrials.gov (identifier: NCT05749731)*.*

### Setting and participants

We used the consensus definition of geriatric rehabilitation published by the EuGMS [6], including facilities which provided multidisciplinary rehabilitation care to frail and/or multimorbid patients. Both inpatient facilities and geriatric rehabilitation at home were included in the EU-COGER consortium (Appendix I).

Geriatric rehabilitation care facilities were recruited from the Czech Republic, Germany, Ireland, Israel, Italy, Malta, the Netherlands, Russia, Spain, and the United Kingdom by members of the EuGMS Special Interest Group. The Special Interest Group members acted as country coordinators, and maintained contact with local study coordinators in participating geriatric rehabilitation care facilities in their country [11]. Patients admitted to participating facilities were recruited by the local study coordinators, between September 2020 and October 2021. Patients could be included if admitted to recover from a SARS-CoV-2 infection, confirmed with either polymerase chain reaction (PCR) for viral RNA or serology for virus antibodies, depending on local protocols. Severe cognitive impairment which prevented patients from providing consent was an exclusion criterion [12]. In total, 793 patient records were created in the database, of which 70 were excluded due to three centres withdrawing from study participation (*n* = 7), duplicates (*n* = 2), empty records (*n* = 10), and patients who did not meet inclusion criteria (*n* = 51).

### Ethics

The study was performed in accordance with the declaration of Helsinki (2013 version) for medical research and general data protection regulation (GDPR). The Leiden University Medical Center COVID-19 science ethical committee deemed this study exempt from the medical research involving human subjects act (WMO) since the study only used routinely collected data, and approved the study based on an opt-out procedure for the Netherlands (protocol number CoCo 2020–040). In all other countries, the local regulations were adhered to and, when required, additional approval was obtained from a local Ethics committee.

### Data collection

#### Cohort data

Routinely collected medical care data from patients’ (electronic) health records were collected in cloud-based clinical data management system Castor [13]. Data were collected at admission to geriatric rehabilitation, including pre-morbid (pre-COVID) status from referral letters, at geriatric rehabilitation discharge, 6 weeks and 6 months follow-up. A complete overview of data collected is provided in the study protocol [12].

The primary outcome measure was daily functioning, assessed with the Barthel Index for activities of daily living [14]. The Barthel Index by Collin et al. produces a total score of 20, where higher scores represent higher independence in activities of daily living. This is the only functional outcome measure routinely collected across participating countries. Certain countries or facilities used the Utrecht scale for the evaluation of rehabilitation (USER) or the Functional Independence Measure (FIM). These comparable measures were converted to Barthel index using standardized approaches [15, 16]. The secondary outcome measure was health-related quality of life assessed with the EQ-5D-5L, available in over 150 languages [17]*.* The EQ-5D-5L is a 5-item instrument that produces a maximum score of 1 for optimal quality of life. Patients’ EQ-5D-5L scores were calculated using available country tariffs [18–23]. For Malta, Czech Republic, and Russia no country tariffs were available and the geographically closest available country tariffs (Spain, Poland and Poland respectively) were used [21, 24]. In Israel and the United Kingdom no quality of life data were collected as part of routine practice. In addition, duration of geriatric rehabilitation and discharge destination across countries are described in Table 2. Data collected about treatment components provided as part of geriatric rehabilitation comprised: oxygen therapy, physiotherapy, occupational therapy, speech and language therapy, protein or calorie enriched diets, psychosocial support, and cognitive training. The number of missing data is presented in Appendix II.

#### Survey data

During the EU-COGER project, we noticed that there are large differences in characteristics and triage of post-COVID patients across countries that have implications for the health condition of patients at the time of admission to geriatric rehabilitation. Therefore, a survey was developed to collect data about the referral process of post-COVID-19 patients to geriatric rehabilitation and characteristics of geriatric rehabilitation care organization in participating countries. The survey comprised multiple choice and open questions about types of geriatric rehabilitation care facilities, selection criteria for referral for patients recovering from COVID-19 to geriatric rehabilitation, and geriatric rehabilitation discharge criteria for this patient group. The survey is presented in Appendix III and a glossary of GR care facilities is in Appendix IV. The study’s country coordinators answered these questions for the participating care providers from their country.

### Data analysis

#### Cohort data

Patients’ demographic and clinical characteristics and treatment components were analyzed using descriptive statistics. Normally distributed continuous variables were reported with mean and SD, other continuous variables with median and interquartile range (IQR). Categorical variables were presented as percentages (%) and numbers (*n*).

The recovery trajectories of daily functioning and quality of life between admission and discharge from geriatric rehabilitation were examined using linear mixed models, with time operationalized as weeks since admission to geriatric rehabilitation. Linear trajectories were modelled using data from admission to discharge. For the Barthel index two splines were fitted, as the premorbid measurement was also included. Random intercept and random linear slope parameters for variance between participants were added when they improved model fit. Models were built with unstructured variance–covariance matrices. The models had two levels for measurements nested in patients. Country was added as an independent categorical variable, and models were adjusted for mean centred age and sex. This enabled us to plot recovery trajectories for the average participant in each country. All models were built using R package lme4 in R version 4.2.2. Model estimates are presented in Appendix V.

#### Survey data

Responses to multiple choice questions about geriatric rehabilitation facility types, selection criteria for referral to geriatric rehabilitation, and geriatric rehabilitation discharge criteria were converted to tabular form with checkboxes for participating countries. Answers to open questions regarding patient selection and discharge criteria were inductively grouped into categories by TL and checked by LST. These data conversions were checked by the country coordinators, each for the participating care facilities from their country.

## Results

### Patients characteristics

A total of 723 patients from 59 European rehabilitation facilities were included in the analysis. Participating countries were the Czech Republic (*n* = 53), Germany (*n* = 50), Ireland (*n* = 50), Israel (*n* = 32), Italy (*n* = 30), Malta (*n* = 17), the Netherlands (*n* = 293), Russia (*n* = 50), Spain (*n* = 96), and the United Kingdom (*n* = 52) (Table 1).

**Table 1:** Characteristics of post-COVID-19 patients in geriatric rehabilitation (GR)

	All	CZ	DE	IE	IL	IT	MT	NL	RU	ES	UK
Participants, *n* (%)	723 (100)	53 (7.3)	50 (6.9)	50 (6.9)	32 (4.4)	30 (4.1)	17 (2.4)	293 (40.6)	50 (6.9)	96 (13.3)	52 (7.2)
Age, mean (SD)	75.7 (9.8)	79.0 (9.8)	83.1 (6.0)	74.2 (11.0)	81.2 (8.3)	75.6 (7.0)	74.6 (6.3)	73.6 (9.0)	75.2 (7.1)	73.1 (11.3)	81.6 (11.4)
Sex, male, *n* (%)	379 (52.4)	19 (35.8)	19 (38.0)	27 (54.0)	15 (46.9)	25 (83.3)	12 (70.6)	163 (55.6)	16 (32.0)	63 (65.6)	20 (38.5)
Number of comorbidities, FCI, median (IQR)	3.0 (2.0–4.0)	5.0 (3.0–6.0)	3.0 (2.0–4.0)	3.0 (1.0–4.0)	3.0 (2.0–4.0)	3.0 (1.0–4.0)	2.0 (1.0–3.75)	2.0 (1.0–4.0)	6.0 (5.0–8.0)	3.0 (1.0–4.0)	3.0 (2.0–4.0)
Frailty at GR admission, CFS, median (IQR)	6.0 (5.0–7.0)	6.0 (5.0–6.0)	6.0 (4.0–6.0)	6.0 (6.0–7.0)	NAMP^	4.0 (4.0–5.5)	7.0 (6.0–7.0)	NAMP^	3.5 (3.0–4.25)	6.0 (5.0–7.0)	7.0 (6.0–7.0)
Lived at home premorbid, *n* (%)	675 (93.4)	47 (88.7)	46 (92.0)	50 (100.0)	30 (93.8)	30 (100.0)	16 (94.1)	287 (98.0)	27 (54.0)	94 (97.9)	48 (92.3)
Hospital stay before GR admission	653 (90.3)	45 (84.9)	49 (98.0)	41 (82.0)	32 (100.0)	30 (100.0)	16 (94.1)	267 (91.1)	28 (56.0)	95 (99.0)	50 (96.2)
Hospital length of stay prior to GR, days, median (IQR)	23.0 (13.0–46.5)	13.0 (7.0–23.0)	28.0 (18.0–51.5)	26.0 (15.0–52.0)	29.0 (20.0–33.0)	53.0 (39.5–65.25)	51.0 (30.25–66.0)	21.0 (13.0–40.0)	10.0 (6.25–14.75)	40.0 (18.0–63.5)	16.5 (9.0–63.25)
ICU stay prior to GR, *n* (%)	240 (33.2)	9 (17.0)	11 (22.0)	14 (28.0)	9 (28.1)	4 (13.3)	11 (64.7)	118 (40.3)	3 (6.0)	54 (56.3)	7 (13.5)

*CZ* the Czech Republic, *DE* Germany, *IE* Ireland, *IL* Israel, *IT* Italy, *MT* Malta, *NL* the Netherlands, *RU* Russia, *ES* Spain, *UK* the United Kingdom, *FCI* functional comorbidity index, *CFS* clinical frailty scale; ^*NAMP* not available for majority of patients

Patient characteristics varied between countries. Mean age was 75.7 years (SD 9.9), ranging from 73.1 (SD 11.3) in Spain and 73.6 (SD 9.0) in the Netherlands to 83.1 (SD 6.0) years old in Germany (Table 1). The percentage of male participants ranged from 32.0% in Russia, 35.8% in the Czech Republic, and 38.0% in Germany, to 83.3% in Italy. Participants’ median FCI score for comorbidities was 3.0 (IQR 2.0–4.0). Participants seemed to have fewer comorbidities in Malta (2.0, IQR 1.0–3.8) and the Netherlands (2.0, IQR 1.0–4.0), and seemed to have more comorbidities in the Czech Republic (5.0, IQR 2.0–6.0) and Russia (6.0, IQR 5.0–8.0).

Most participants (93.4%) lived at home before SARS-CoV-2 infection. In Ireland and Italy this was the case for all participants. In Russia, much smaller percentages of participants lived at home before infection (54.0%); almost half of the Russian participants (46.0%) lived in a nursing home. In all countries except Russia, over 80% of patients had been admitted to the hospital due to COVID-19 before admission to geriatric rehabilitation (in total 90.3%). There was a variation in the median duration of this hospital stay, from 53 days in Italy (IQR 39.5–65.25) and 51 days in Malta (IQR 30.25–66.0), to only 13 days in the Czech Republic (7.0–23.0) and 10 days in Russia (6.25–14.75). One-third of the patients (33.2%) had stayed at an intensive care unit (ICU), but these percentages were much lower in the Czech Republic (17%), Germany (22%), Italy (13.3%), Russia (6%) and the United Kingdom (13.5%), and much higher in Malta (64.7%) and Spain (56.3%).

The mean Barthel Index score at admission to geriatric rehabilitation for participants from all countries was 10.9 (SD 5.4) (Table 2), and most participants were living with moderate frailty (median CFS 6.0, IQR 5.0–7.0) (Table 1). In Italy and Russia, the Barthel Index scores of the participants at admission were more than two points higher than the cohort’s mean (15.5, SD 3.9; 16.1, SD 4.0, respectively) and participants seemed to be less frail than in other countries (median CFS 4.0, IQR 4.0–5.5; median CFS 3.5, IQR 3.0–4.25, respectively). In Israel, Malta, and the United Kingdom, Barthel Index scores at admission were more than two points lower than the cohort’s mean (4.0, SD 2.2; 6.3, SD 3.5; 6.4, SD 4.7, respectively) and participants seemed to be frailer than in other countries (median CFS 6.0, IQR 6.0–7.0; median CFS 7.0, IQR 6.0–7.0; median CFS 7.0, IQR 6.0–7.0, respectively). In Ireland and the United Kingdom, the EQ-5D-5L scores of the participants at admission (0.26, SD 0.40; 0.28 SD 0.06, respectively) seemed to be lower than the cohort’s mean (0.52, SD 0.32).

**Table 2:** Outcomes of post-COVID-19 patients in geriatric rehabilitation (GR)

	All	CZ	DE	IE	IL	IT	MT	NL	RU	ES	UK
Participants, *n* (%)	723 (100)	53 (7.3)	50 (6.9)	50 (6.9)	32 (4.4)	30 (4.1)	17 (2.4)	293 (40.6)	50 (6.9)	96 (13.3)	52 (7.2)
Duration GR, weeks, median (IQR)	3.7 (2.1–5.7)	3.1 (2.2–3.9)	2.9 (2.7–3.9)	3.4 (1.5–5.8)	3.4 (2.4–6.2)	4.9 (4.1–5.6)	6.4 (3.4–7.5)	4.1 (2.7–6.6)	1.9 (1.4–2.0)	4.7 (3.4–6.4)	3.1 (2.0–4.7)
Daily functioning, Barthel index, mean (SD)											
at GR admission	10.9 (5.4)	10.7 (3.7)	12.7 (3.8)	10.6 (4.1)	4.0 (2.2)	15.5 (3.9)	6.2 (3.5)	11.9 (5.0)	16.1 (4.0)	9.0 (5.6)	6.4 (4.7)
at GR discharge	15.9 (4.7)	15.4 (3.0)	16.5 (2.9)	16.8 (17.0)	10.3 (4.6)	18.1 (2.8)	14.8 (7.1)	17.3 (3.6)	17.1 (3.6)	16.1 (5.0)	9.5 (6.3)
Quality of life, EQ-5D-5L, mean (SD)											
at GR admission	0.52 (0.32)	0.51 (0.24)	0.64 (0.29)	0.26 (0.40)	NAMP^	0.68 (0.15)	0.53 (0.37)	NAMP^	0.85 (0.15)	0.45 (0.31)	0.28 (0.06)
at GR discharge	0.77 (0.22)	0.68 (0.19)	0.81 (0.150	0.61 (0.30)	NAMP^	0.86 (0.11)	0.85 (0.20)	NAMP^	0.91 (0.11)	0.78 (0.24)	NAMP^
Discharge destination, *n* (%)											
Own home	544 (75.2)	22 (41.5)	37 (74.0)	41 (82.0)	21 (65.6)	27 (90.0)	14 (82.4)	246 (84.0)	25 (50.0)	78 (81.3)	33 (63.5)
Assisted living	20 (2.8)	5 (9.4)	2 (4.0)	0 (0.0)	2 (6.3)	0 (0)	0 (0.0)	6 (2.0)	0 (0.0)	5 (5.2)	0 (0)
Nursing home	83 (11.5)	20 (37.7)	6 (12.0)	0(0.0)	7 (21.9)	1 (3.3)	1 (5.9)	7 (2.4)	24.0 (48.0)	5 (5.2)	12 (23.1)
Hospital	30 (4.1)	1 (1.9)	3 (6.0)	1 (2.0)	1 (3.1)	2 (6.7)	1 (5.9)	11 (3.8)	1 (2.0)	3 (3.1)	6 (11.5)
Deceased during GR	11 (1.5)	0 (0)	0 (0.0)	2 (4.0)	0 (0.0)	0 (0.0)	0 (0.0)	7 (2.4)	0 (0.0)	2 (2.1)	0 (0.0)
Other	15 (2.1)	5 (9.4)	0(0.0)	4 (8.0)	1 (3.1)	0 (0.0)	1 (5.9)	1 (0.3)	0 (0.0)	2 (2.1)	1 (1.9)

*CZ* the Czech Republic, *DE* Germany, *IE* Ireland, *IL* Israel, *IT* Italy, *MT* Malta, *NL* the Netherlands, *RU* Russia, *ES* Spain, *UK* the United Kingdom, *FCI* functional comorbidity index, *CFS* clinical frailty scale, ^*NAMP* not available for majority of patients

### Referral of post-COVID-19 patients to geriatric rehabilitation

In all countries, a combination of multiple criteria was used when selecting patients for geriatric rehabilitation, but there was substantial heterogeneity in which criteria were used between countries. In the Irish and Italian facilities, two criteria were used for patient selection (cognitive status and functional status, and cognitive status and psychosocial needs respectively). In facilities from other countries combinations of up to six criteria were used (Table 3).

**Table 3:** Post-COVID-19 geriatric rehabilitation (GR): selection criteria, types of care facilities, and discharge criteria in the participating facilities

	All	CZ	DE	IE	IL	IT	MT	NL	RU	ES	UK
Participants, *n* (%)	723 (100)	53 (7.3)	50 (6.9)	50 (6.9)	32 (4.4)	30 (4.1)	17 (2.4)	293 (40.6)	50 (6.9)	96 (13.3)	52 (7.2)
GR selection criteria, x = criterion used											
Minimum age cut-off		65 +	Varying	Varying	65 +	Varying	60 +	N/A	65 +	N/A	Varying
Comprehensive geriatric assessment			x				x	x	x	x	x
Functional status		x	x	x	x		x	x	x	x	x
Frailty level		x			x		x	x	x	x	x
Multimorbidity			x		x			x	x	x	x
Psychosocial needs					x	x		x	x	x	
Cognitive impairment		x	x	x	x	x		x			x
GR care facilities, x = included facility type											
Nursing home/LTC facility										x	x
Skilled nursing facility								x			
Acute care hospital ward		x		x						x	x
Specialized rehabilitation facility		x	x	x	x	x	x	x	x	x	
Intermediate care facility										x	x
Home-based treatment								x			x
Ambulatory/outpatient treatment								x	x	x	
GR discharge criteria, x = criterion used											
Ability to function in the premorbid living situation (with or without support)		x		x	x		x		x		
Duration of GR		Varying	21 days	N/A	90 days	30 days	N/A	180 days	60 days	N/A	42 days
Achievement of personal goals								x			x
Stability (medically/rehabilitation)								x		x	

*CZ* the Czech Republic, *DE* Germany, *IE* Ireland, *IL* Israel, *IT* Italy, *MT* Malta, *NL* the Netherlands, *RU* Russia, *ES* Spain, *UK* the United Kingdom, *LTC* long-term care

Minimum ages were used as selection criteria for geriatric rehabilitation across eight participating countries but not the Netherlands and Spain. In seven of the ten countries daily functioning and frailty were used as referral criteria. In German and Irish care facilities, daily functioning was used as a criterion but not frailty, and in the Italian care facilities neither one of these characteristics was used. Comorbidities were used as selection criterion in care facilities from six countries, but not in the Czech Republic, Ireland, Italy or Malta. Quality of life was not used as a selection criterion. Comprehensive Geriatric Assessment was used in patient selection for geriatric rehabilitation in six countries. In six countries, cognitive impairment that may affect adherence to geriatric rehabilitation treatment was used as a contraindication for referral to geriatric rehabilitation.

In most countries, selecting patients for geriatric rehabilitation after COVID-19 was the responsibility of hospital physicians and general practitioners (GPs). In Israel, referral could also be done by physio- or occupational therapists or social workers. In the United Kingdom, referral of COVID-19 patients was usually done by hospital nurses and therapists.

### Geriatric rehabilitation care provided to post-COVID-19 patients

Geriatric rehabilitation care was provided in diverse types of care facilities. Participating care facilities included: specialized (geriatric) rehabilitation facilities from all countries except the United Kingdom; long-term care facilities and intermediate care facilities in Spain and the United Kingdom; skilled nursing facilities in the Netherlands; acute care hospital wards in the Czech Republic, Ireland, Spain, and United Kingdom; and geriatric rehabilitation care at home or on ambulatory/outpatient basis in the Netherlands, Russia, and Spain.

**Fig. 1:**
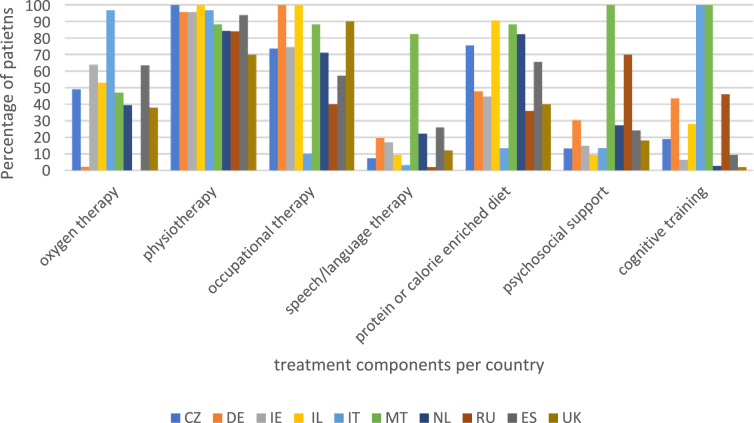
Treatment components of geriatric rehabilitation for post-COVID-19 patients (*n* = 670). *CZ* the Czech Republic, *DE* Germany, *IE* Ireland, *IL* Israel, *IT* Italy, *MT* Malta, *NL* the Netherlands, *RU* Russia, *ES* Spain, *UK* the United Kingdom

Post-COVID-19 geriatric rehabilitation care always comprised various treatments. In all countries at least 70% of participants received physiotherapy. Occupational therapy was provided to at least 70% of the participants in all countries except Italy, Russia, and Spain. Most participants also received protein and calorie enriched diets (65.2%), but these showed greater variance between countries. In the Czech Republic, Israel, Malta, and Netherlands, more than three-quarters of the participants received protein and calorie-enrichment, but in Italy, Russia, and the United Kingdom this was 13.3, 36.0, and 40.0%, respectively (Fig. 1). Large differences between countries were observed in the number of participants receiving oxygen therapy during geriatric rehabilitation (43.1%), from no participants in Russia and only one in Germany, to all but one participant in Italy. Smaller percentages of participants received speech and language therapy (18.8%), psychosocial support (25.4%) and cognitive training (12.2%). In Malta, speech and language therapy was provided much more often than in other countries (82.4%). Psychosocial support was provided more often to participants from Malta and Russia (100.0% and 70.0%, respectively) than in other countries, and cognitive training was provided more often to participants from Italy, Malta, and Russia (100.0%, 100.0%, and 46.0%, respectively) than to participants from other countries.

The median duration of geriatric rehabilitation trajectories was 3.7 weeks (IQR 2.1–5.7). The longest median duration was in Malta (6.4 weeks, IQR 3.4–7.5) and the shortest in Russia (1.9 weeks, IQR 1.4 – 2.0).

### Recovery of post-COVID-19 patients during geriatric rehabilitation

**Fig. 2:**
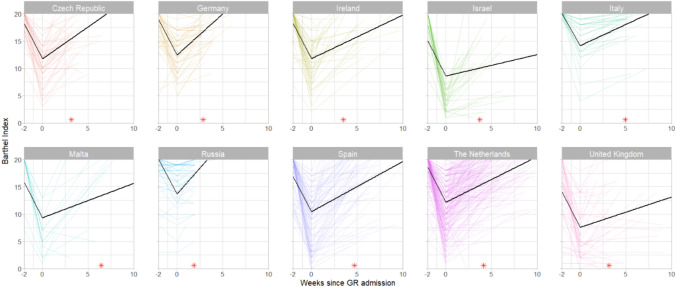
Recovery of post-COVID-19 patients in daily functioning during geriatric rehabilitation (GR) and median duration of GR (*)

**Fig. 3:**
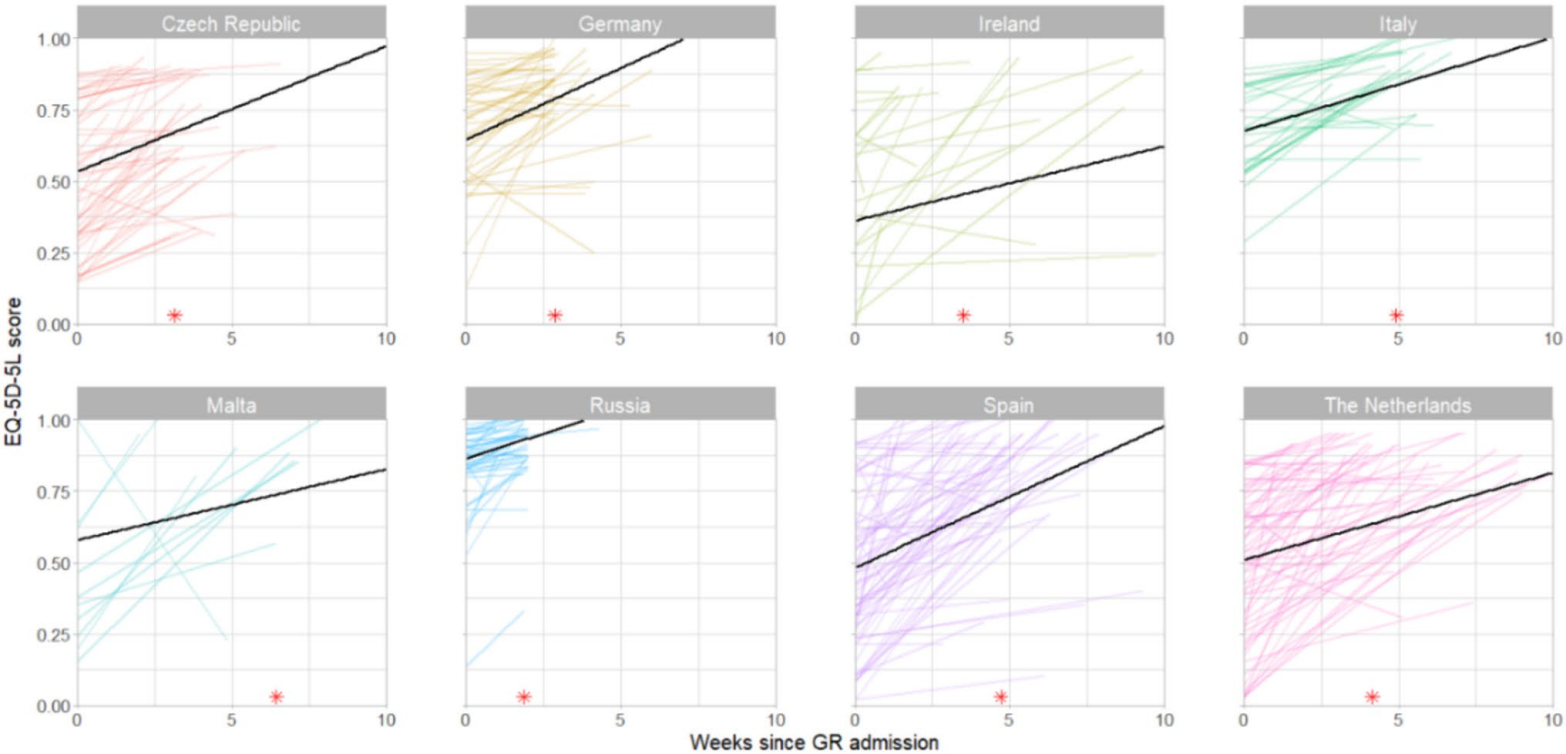
Recovery of post-COVID-19 patients in quality of life during geriatric rehabilitation (GR) and median duration of GR (*)

The recovery trajectories of daily functioning and quality of life in each country are shown in Figs. 2 and 3, respectively. In all countries, participants’ Barthel Index scores decreased from premorbid to admission to geriatric rehabilitation and increased again during geriatric rehabilitation. These increases were the steepest in participants from the Czech Republic, Germany, and Russia, and the least steep in Israel, the United Kingdom and Malta (Appendix V). On average, participants did not reach their premorbid Barthel Index score during geriatric rehabilitation. Due to heterogeneous Barthel Index scores at admission, countries with the largest increase in Barthel Index between admission and discharge (Malta and Spain) were not the same as those with the highest Barthel Index score at discharge (Netherlands and Italy). Like Barthel Index scores, EQ-5D-5L scores increased in all countries where these were measured during geriatric rehabilitation. The steepest increases were in participants from the Czech Republic, Germany and Spain, and the least steep in Ireland, Malta, and the Netherlands (Appendix V).

### Discharge of post-COVID-19 patients from geriatric rehabilitation

Table 3 indicates which criteria were used to determine discharge from geriatric rehabilitation by country. In several countries, participants were discharged when they were able to function independently (or with help of family) and/or to go back to their premorbid living situation. Besides this, achievement of personal treatment goals and stabilization of medical condition were criteria for discharge. In six of the ten countries, post-COVID-19 patients were automatically discharged after maximum duration of geriatric rehabilitation, ranging from 3.0 weeks (21 days) in participating German care facilities to 25.7 weeks (180 days) in facilities in the Netherlands. In Germany and in Italy, these permitted maximum durations (3 weeks (21 days) and 4.3 weeks (30 days), respectively) were exceeded by some participants (IQR 19.0–27.0; and IQR 29.0 – 39.0 days, respectively).

When discharged from geriatric rehabilitation, 75.2% of all study participants returned home (more than 65% in seven countries), compared to 93.4% still living at home premorbidly. However, in the Czech Republic, where patients’ daily functioning recovery rates were relatively high, less than half (41.5%, n = 22) of the 47 participants that premorbidly lived at home (88.7%) returned home after rehabilitation.

## Discussion

This study provides insight into the selection criteria for referral to geriatric rehabilitation, care provided, and post-COVID-19 recovery in patients receiving geriatric rehabilitation across ten different European countries. Across countries, post-COVID-19 patients showed recovery in daily functioning and quality of life during geriatric rehabilitation. The variation in selection criteria and patient characteristics was accompanied by some variation in recovery outcomes. All participating European countries used multiple selection criteria to refer patients to geriatric rehabilitation, often including patients’ functional status, age, frailty, CGA, comorbidities, and cognitive impairments. Although care settings and care provided varied widely, in all countries the majority of patients received physiotherapy, and in many countries the majority also received occupational therapy. The median duration of geriatric rehabilitation trajectories ranged from 13 to 45 days across countries. In all countries post-COVID-19 patients showed recovery in daily functioning and quality of life during geriatric rehabilitation, albeit at variable rates. The steepest increases in daily functioning were seen in the Czech Republic, Germany, and Russia, and the steepest increases in quality of life were seen in the Czech Republic, Germany, and Spain.

Geriatric rehabilitation care already varied across Europe prior to the pandemic. Previous studies described large differences in care settings in which geriatric rehabilitation care is provided [7], duration of geriatric rehabilitation trajectories, and geriatric rehabilitation capacity [8]. Despite these differences in settings, physiotherapists and occupational therapists have been the practitioners that were most often involved in geriatric rehabilitation teams across Europe [8] and this is reflected in our data. Variation in geriatric rehabilitation care between countries may have further increased during the pandemic because of differences in infection rates and ways of coping with COVID-19 [25]. In Europe, Italy and the United Kingdom were hit hard during the first wave (spring 2020), with the highest death rates per million population [26]. In the United Kingdom, rates of staff absenteeism also increased from 4 to over 6% during the pandemic [27]. Moreover, the impact of the pandemic on the United Kingdom’s healthcare system might have been even more severe due to pre-existing vulnerability before the pandemic, as the number of hospital beds and medical staff [28] per capita was already low. This seems to be in line with the relatively low recovery rate of daily functioning which we observed in the United Kingdom.

Based on the descriptive results of this study, hypotheses can be generated about potential relationships between patient characteristics, selection criteria, organizational aspects of geriatric rehabilitation, and recovery. Some remarkable things in our data are, for example, first, that two of the three countries with the steepest recovery in daily functioning, the Czech Republic and Russia, are also the only countries that maintained a relatively high minimum age of 65 years old for geriatric rehabilitation selection. However, the mean ages of Czech and Russian patients (respectively, 79.0 and 75.2) did not differ much from the population mean (75.7). In line with our findings, it has been suggested that age criteria should be combined with, for example, frailty criteria [7]. Second, in countries with the lowest recovery rates in daily functioning, i.e., Israel, Malta, and the United Kingdom, patients were more frail and had lower daily functioning levels at admission to geriatric rehabilitation than in other countries. However, a previous publication of the EU-COGER study shows that post-COVID-19 patients who are frail at admission to geriatric rehabilitation also have the potential to substantially recover in daily functioning [29]. Third, in the three countries with the highest recovery rates in daily functioning, the Czech Republic, Germany, and Russia, the lowest percentages of male participants were observed and few patients stayed at an ICU prior to admission to geriatric rehabilitation. It is also described in literature that for male COVID-19 patients outcomes are worse than for female patients [30], and that for other patient groups’ recovery after an ICU stay is difficult [31]. However, in our study, large heterogeneity in known and unknown organizational variables may also have influenced recovery. Fourth, regarding treatment components, patients from countries with high recovery rates, such as the Czech Republic and Germany, mainly received physiotherapy and occupational therapy, and in the Czech Republic also protein or calorie enriched diets. This suggests that physiotherapy, occupational therapy, and protein or calorie enriched diets are most important to increase daily functioning and quality of life. However, before recommendations can be made about how to optimize geriatric rehabilitation for post-COVID-19 patients, future explanatory research should confirm which organizational aspects of geriatric rehabilitation and which patient characteristics affect recovery. To do so, measurements of patient characteristics and recovery outcomes in geriatric rehabilitation across countries should be harmonized.

Some limitations of this study should be recognized. Firstly, we collected data from only a limited number of care facilities per country, and in some countries from small numbers of patients. Especially in large countries with small sample sizes, our data are unlikely to be representative of the participating countries as a whole. Secondly, the collected data may not provide a complete reflection of the geriatric rehabilitation care provided across Europe, as the treatment components presented in this study are not exhaustive. The most relevant treatment variables were, however, included as the study was designed by members of the EuGMS special interest group for geriatric rehabilitation from different countries, who can be regarded as experts in the field. Thirdly, as a consequence of only collecting routine care data, more detailed outcome measures of recovery (e.g. iADL), information about the frequency and duration of geriatric rehabilitation treatment components are lacking, and the survey did not collect cut-off values in selection and discharge criteria. Insight into these factors could add to a better understanding of rehabilitation and recovery in each country [32]. Fourthly, the observed variation in recovery rates may partly be explained by variation in the timing of patients’ admission to geriatric rehabilitation. During the inclusion period, between September 2020 and October 2021, treatment effectiveness and organization of geriatric rehabilitation care for post-COVID-19 patients might have improved. It would be interesting to conduct future research into changes in care over time.

A strength of this study is that patients from care facilities from ten European countries were included. This international collaboration was set up quickly during a turbulent time. This study provides unique insight into the care provided on a large scale during the first year of the pandemic. A second strength is the combination of patient data from our cohort with survey data about the care organization in participating countries. This provides a comprehensive picture of post-COVID-19 patients admitted to geriatric rehabilitation, their recovery during geriatric rehabilitation, and the organization of geriatric rehabilitation care. A third strength is that this study focused on geriatric rehabilitation after acute COVID-19. Although a number of studies have observed recovery of older COVID-19 patients, not much research has been focused on rehabilitation [33].

## Conclusion

The present study shows that post-COVID-19 patients substantially recover during geriatric rehabilitation across Europe, although there was variation in the rates of recovery between countries. This variation may partly be explained by the heterogeneity in geriatric rehabilitation practice and patient characteristics between countries. This heterogeneity complicates international comparisons. Moreover, this heterogeneity suggests that geriatric rehabilitation has not been given equal priority between countries. This study may enable countries to learn from each other, and facilitated the generation of hypotheses about factors that are related to recovery. Future explanatory studies and harmonisation of measurements in geriatric rehabilitation are needed to understand the optimal configuration of rehabilitation care. The ultimate goal should be to ensure that all patients, wherever they live, can receive the best available rehabilitation care to which they are entitled.

## Supplementary Information

Below is the link to the electronic supplementary material.

Supplementary file1: Appendix III (PDF 576 KB)

Supplementary file2: Appendices I, II, IV, V, VI (DOCX 45 KB)

## Data Availability

The data are not publicly available due to the agreement with participating care facilities and the consent provided by patients included. Researchers who wish to conduct analyses using EU-COGER data should submit a proposal to P.I. Prof. Wilco Achterberg (W.P.Achterberg@lumc.nl) including research questions and an analysis plan. If the request is approved, a data transfer agreement has to be signed before the data will be shared.
