# Supplementary material for: Post-COVID-19 recovery and geriatric rehabilitation care: a European inter-country comparative study
Source: Eur Geriatr Med. 2024 Aug 13;15(5):1489–501. doi: 10.1007/s41999-024-01030-w (PMC11614975; doi:10.1007/s41999-024-01030-w)
Supplement: Supplementary file 1 — Supplementary file1: Appendix III (PDF 576 KB) [file 41999_2024_1030_MOESM1_ESM.pdf]

Surveys of EU-COGER rehab survey - version 52.91

Printed on 09-09-2021 22:37:24 by Wendy Meester

Survey 'EU-COGER rehab survey'

EU-COGER rehab survey - survey_correct

Number	Question	Answers
	About you	
1.1	Which country do you represent	◯ Afghanistan<br>◯ Albania<br>◯ Algeria<br>◯ Andorra<br>◯ Angola<br>◯ Antigua and Barbuda<br>◯ Argentina<br>◯ Armenia<br>◯ Australia<br>◯ Austria<br>◯ Azerbaijan<br>◯ Bahamas<br>◯ Bahrain<br>◯ Bangladesh<br>◯ Barbados<br>◯ Belarus<br>◯ Belgium<br>◯ Belize<br>◯ Benin<br>◯ Bhutan<br>◯ Bolivia<br>◯ Bosnia and Herzegovina<br>◯ Botswana<br>◯ Brazil<br>◯ Brunei<br>◯ Bulgaria<br>◯ Burkina Faso<br>◯ Burundi<br>◯ Cabo Verde<br>◯ Cambodia<br>◯ Cameroon<br>◯ Canada<br>◯ Central African Republic

- Chad
- Chile
- China
- Colombia
- Comoros
- Democratic Republic of the Congo
- Republic of the Congo
- Costa Rica
- Cote d Ivoire
- Croatia
- Cuba
- Cyprus
- Czech Republic
- Denmark
- Djibouti
- Dominica
- Dominican Republic
- Ecuador
- Egypt
- El Salvador
- Equatorial Guinea
- Eritrea
- Estonia
- Ethiopia
- Fiji
- Finland
- France
- Gabon
- Gambia
- Georgia
- Germany
- Ghana
- Greece
- Grenada
- Guatemala
- Guinea
- Guinea-Bissau
- Guyana
- Haiti
- Honduras
- Hungary
- Iceland
- India
- Indonesia
- Iran
- Iraq
- Ireland

- ○ Israel
- ○ Italy
- ○ Jamaica
- ○ Japan
- ○ Jordan
- ○ Kazakhstan
- ○ Kenya
- ○ Kiribati
- ○ Kosovo
- ○ Kuwait
- ○ Kyrgyzstan
- ○ Laos
- ○ Latvia
- ○ Lebanon
- ○ Lesotho
- ○ Liberia
- ○ Libya
- ○ Liechtenstein
- ○ Lithuania
- ○ Luxembourg
- ○ Macedonia
- ○ Madagascar
- ○ Malawi
- ○ Malaysia
- ○ Maldives
- ○ Mali
- ○ Malta
- ○ Marshall Islands
- ○ Mauritania
- ○ Mauritius
- ○ Mexico
- ○ Micronesia
- ○ Moldova
- ○ Monaco
- ○ Mongolia
- ○ Montenegro
- ○ Morocco
- ○ Mozambique
- ○ Myanmar (Burma)
- ○ Namibia
- ○ Nauru
- ○ Nepal
- ○ Netherlands
- ○ New Zealand
- ○ Nicaragua
- ○ Niger
- ○ Nigeria

- North Korea
- Norway
- Oman
- Pakistan
- Palau
- Palestine
- Panama
- Papua New Guinea
- Paraguay
- Peru
- Philippines
- Poland
- Portugal
- Qatar
- Romania
- Russia
- Rwanda
- Saint Kitts and Nevis
- Saint Lucia
- Saint Vincent and the Grenadines
- Samoa
- San Marino
- Sao Tome and Principe
- Saudi Arabia
- Senegal
- Serbia
- Seychelles
- Sierra Leone
- Singapore
- Slovakia
- Slovenia
- Solomon Islands
- Somalia
- South Africa
- South Korea
- South Sudan
- Spain
- Sri Lanka
- Sudan
- Suriname
- Swaziland
- Sweden
- Switzerland
- Syria
- Taiwan
- Tajikistan
- Tanzania

		◯ Thailand<br>◯ Timor-Leste<br>◯ Togo<br>◯ Tonga<br>◯ Trinidad and Tobago<br>◯ Tunisia<br>◯ Turkey<br>◯ Turkmenistan<br>◯ Tuvalu<br>◯ Uganda<br>◯ Ukraine<br>◯ United Arab Emirates<br>◯ United Kingdom<br>◯ United States of America<br>◯ Uruguay<br>◯ Uzbekistan<br>◯ Vanuatu<br>◯ Vatican City<br>◯ Venezuela<br>◯ Vietnam<br>◯ Yemen<br>◯ Zambia<br>◯ Zimbabwe
1.2	At which institution do you work?	
	About post-acute care in general in your country	
1.3	What are the different options for post-acute care in your country	☐ Institutionalized treatment and care from a single discipline<br>☐ Institutionalized treatment and care from a multidisciplinary team<br>☐ Ambulatory treatment and care from a single discipline<br>☐ Ambulatory treatment and care from a multidisciplinary team<br>☐ Home-based treatment and care from a single discipline<br>☐ Home-based treatment and care from a multidisciplinary team<br>☐ Other<br>☐ I don't know this
1.3.1	***If 'What are the different options for post-acute care in your country' is equal to 'Other' answer this question:*** Please indicate what you mean with 'other':	

	About geriatric rehabilitation (GR) in your country	
1.4	Where is GR provided in your country	☐ At a nursing home/long term care facility<br>☐ At a skilled nursing facility<br>☐ At an acute care hospital ward<br>☐ At a specialised (geriatric) rehab facility<br>☐ At an intermediate care facility<br>☐ At home<br>☐ On outpatient basis<br>☐ Other<br>☐ I don't know this
1.4.1	***If 'Where is GR provided in your country' is equal to 'Other' answer this question:***<br>Please indicate what you mean with 'other':	
	The following questions relate specifically to post-COVID patients	
1.5	What type of facilities are participating in the EU-COGER study on behalf of your country?	☐ A nursing home/long term care facility<br>☐ A skilled nursing facility<br>☐ An acute care hospital ward<br>☐ A specialised (geriatric) rehab facility<br>☐ An intermediate care facility<br>☐ Facilities providing care at home<br>☐ Faciltities providing care on outpatient basis<br>☐ Other<br>☐ I don’t know this
1.5.1	***If 'What type of facilities are participating in the EU-COGER study on behalf of your country?' is equal to 'Other' answer this question:***<br>Please indicate what you mean with 'other':	
1.6	Where is GR for post-COVID patients provided in your country? (please indicate all applicable facility types, also including those that are currently NOT represented in the EU-COGER study)	☐ At a nursing home/long term care facility<br>☐ At a skilled nursing facility<br>☐ At an acute care hospital ward<br>☐ At a specialised (geriatric) rehab facility<br>☐ At an intermediate care facility<br>☐ At home<br>☐ On outpatient basis<br>☐ Other<br>☐ I don't know this

1.6.1	***If 'Where is GR for post-COVID patients provided in your country? (please indicate all applicable facility types, also including those that are currently NOT represented in the EU-COGER study)' is equal to 'Other' answer this question:***<br>Please indicate what you mean with 'other':		
	The following questions with sliders are about the distribution of post-COVID patients over the nationally available facilities. Please give an estimation of the number. E.g. if 20% of post-COVID GR in your country was provided at home, then you put the slider for "at home" to 20%		
1.6.2	***If 'Where is GR for post-COVID patients provided in your country? (please indicate all applicable facility types, also including those that are currently NOT represented in the EU-COGER study)' is equal to 'At a nursing home/long term care facility' answer this question:***<br>Percentage of post-COVID GR provided at a nursing home/long term care facility	0% (0.00)	100% (100.00) %
1.6.3	***If 'Where is GR for post-COVID patients provided in your country? (please indicate all applicable facility types, also including those that are currently NOT represented in the EU-COGER study)' is equal to 'At a skilled nursing facility' answer this question:***<br>Percentage of post-COVID GR provided at a skilled nursing facility	0% (0.00)	100% (100.00) %
1.6.4	***If 'Where is GR for post-COVID patients provided in your country? (please indicate all applicable facility types, also including those that are currently NOT represented in the EU-COGER study)' is equal to 'At an acute care hospital ward' answer this question:***<br>Percentage of post-COVID GR provided at an acute care hospital ward	0% (0.00)	100% (100.00) %
1.6.5	***If 'Where is GR for post-COVID patients provided in your country? (please indicate all applicable facility types, also including those that are currently NOT represented in the EU-COGER study)' is equal to 'At a specialised (geriatric) rehab facility' answer this question:***<br>Percentage of post-COVID GR provided at a specialised (geriatric) rehab facility	0% (0.00)	100% (100.00) %
1.6.6	***If 'Where is GR for post-COVID patients provided in your country? (please indicate all applicable facility types, also including those that are currently NOT represented in the EU-COGER study)' is equal to 'At an intermediate care facility' answer this question:***<br>Percentage of post-COVID GR provided at an intermediate care facility	0% (0.00)	100% (100.00) %
1.6.7	***If 'Where is GR for post-COVID patients provided in your country? (please indicate all applicable facility types, also including those that are currently NOT represented in the EU-COGER study)' is equal to 'At home' answer this question:***<br>Percentage of post-COVID GR provided at home	0% (0.00)	100% (100.00) %

1.6.8	***If 'Where is GR for post-COVID patients provided in your country? (please indicate all applicable facility types, also including those that are currently NOT represented in the EU-COGER study)' is equal to 'On outpatient basis' answer this question:***<br>Percentage of post-COVID GR provided on outpatient basis	0%<br>(0.00)	100%<br>(100.00)<br>%
1.6.9	***If 'Where is GR for post-COVID patients provided in your country? (please indicate all applicable facility types, also including those that are currently NOT represented in the EU-COGER study)' is equal to 'Other' answer this question:***<br>Percentage of post-COVID GR provided at an 'other' location	0%<br>(0.00)	100%<br>(100.00)<br>%
1.6.10	***If 'Where is GR for post-COVID patients provided in your country? (please indicate all applicable facility types, also including those that are currently NOT represented in the EU-COGER study)' is not equal to 'I don't know this' answer this question:***<br>Total percentage of question 1.6		
1.7	Which person refers the post-COVID patients to GR, i.e. who sets the medical indication/decides ? (e.g. a specific physician in the hospital, the GP, or someone else?).		
1.8	At which moment does the referral to GR for post-COVID patients take place?	☐ After discharge from the acute hospital<br>☐ A few days before discharge from the acute hospital<br>☐ After discharge from the ICU<br>☐ After discharge from other post-acute care facility<br>☐ After suffering from COVID infection at home<br>☐ Other<br>☐ I don't know this<br>☐ Not applicable	
1.8.1	***If 'At which moment does the referral to GR for post-COVID patients take place?' is equal to 'Other' answer this question:***<br>Please indicate what you mean with 'other':		
	The next questions address the selection criteria which need to be met by the post-COVID patient in order to be referred to GR		

1.9	Which patient characteristic is the referral based on?	☐ Age<br>☐ Frailty level<br>☐ Multimorbidity<br>☐ Functional status<br>☐ Psychosocial needs<br>☐ Other<br>☐ I don't know this<br>☐ Not applicable
1.9.1	***If 'Which patient characteristic is the referral based on?' is equal to 'Other' answer this question:***<br>Please indicate what you mean with 'other':	
1.9.2	***If 'Which patient characteristic is the referral based on?' is equal to 'Age' answer this question:***<br>Is there a standardized age cut-off that is used for referral to GR?	○ Yes<br>○ no<br>○ the age cut-off varies by facility<br>○ I don't know this
1.9.2.1	***If 'Is there a standardized age cut-off that is used for referral to GR?' is equal to 'Yes' answer this question:***<br>What is the standardized age cut-off for referral to GR	
1.9.3	***If 'Which patient characteristic is the referral based on?' is equal to 'Frailty level' answer this question:***<br>Which questionnaires/instruments are used to assess frailty level at the moment of referral to GR?	
1.9.4	***If 'Which patient characteristic is the referral based on?' is equal to 'Multimorbidity' answer this question:***<br>Which questionnaires/instruments are used to assess multimorbidity at the moment of referral to GR?	
1.9.5	***If 'Which patient characteristic is the referral based on?' is equal to 'Functional status' answer this question:***<br>Which questionnaires/instruments are used to assess functional status at the moment of referral to GR?	
1.9.6	***If 'Which patient characteristic is the referral based on?' is equal to 'Psychosocial needs' answer this question:***<br>Which questionnaires/instruments are used to assess psychosocial needs at the moment of referral to GR?	

1.9.7	***If 'Which patient characteristic is the referral based on?' is equal to 'Other' answer this question:***<br>Which questionnaires/instruments are used to assess the 'other' criteria at the moment of referral to GR?	
1.10	Which formal assessment is used for GR referral decisions?	☐ Comprehensive geriatric assessment<br>☐ Other<br>☐ Not applicable<br>☐ I don't know this
1.10.1	***If 'Which formal assessment is used for GR referral decisions?' is equal to 'Other' answer this question:***<br>Please indicate what you mean with 'other':	
1.11	Are there contraindications which deem a patient not eligible for post-COVID GR?	◯ Yes<br>◯ No<br>◯ I don't know this
1.11.1	***If 'Are there contraindications which deem a patient not eligible for post-COVID GR?' is equal to 'Yes' answer this question:***<br>Which contraindications deem a patient not eligible for post-COVID GR?	
1.12	Are there any additional criteria being used to determine whether a COVID-19 patient is eligible for referral/transfer to GR?<br>E.g. In certain cases, patients need to be independent of oxygen treatment before they can be transferred to GR.	◯ Yes<br>◯ No<br>◯ I don't know this
1.12.1	***If 'Are there any additional criteria being used to determine whether a COVID-19 patient is eligible for referral/transfer to GR?' is equal to 'Yes' answer this question:***<br>which additional selection criteria are used to determine whether a COVID-19 patient is eligible for referrel/transfer to GR?	
1.13	Are there any selection criteria imposed by health insurance rules/policies for reimbursement of post-COVID GR?	◯ Yes<br>◯ No<br>◯ I don't know this
1.13.1	***If 'Are there any selection criteria imposed by health insurance rules/policies for reimbursement of post-COVID GR?' is equal to 'Yes' answer this question:***<br>Which selection criteria are imposed by health insurance rules/policies for reimbursement of post-COVID GR?	

1.14	Do the GR facilities participating in the EU-COGER study use any additional selection criteria to assess which post-COVID patients are admitted for GR?<br>E.g. do some facilities only admit a certain group of patients for financial reasons?	◯ Yes<br>◯ No<br>◯ I don't know this
1.14.1	***If 'Do the GR facilities participating in the EU-COGER study use any additional selection criteria to assess which post-COVID patients are admitted for GR?' is equal to 'Yes' answer this question:***<br>Which additional selection criteria do they use?	
1.15	Do other GR facilities (i.e. not participating in EU-COGER) in your country use any additional selection criteria to assess which post-COVID patients are admitted for GR?	◯ Yes<br>◯ No<br>◯ I don't know this
1.15.1	***If 'Do other GR facilities (i.e. not participating in EU-COGER) in your country use any additional selection criteria to assess which post-COVID patients are admitted for GR?' is equal to 'Yes' answer this question:***<br>Which additional selection criteria are being used by those other facilities?	
	Questions regarding discharge from GR	
1.16	Which criteria need to be met by post-COVID patients for discharge from GR? And are these criteria standardized nationally/across facilities?	
1.17	Is there a nationally standardized maximum rehabilitation length after which a patient is automatically discharged from GR?	◯ Yes<br>◯ No, there is no maximum length<br>◯ No, the maximum length varies by facility<br>◯ I don't know
1.17.1	***If 'Is there a nationally standardized maximum rehabilitation length after which a patient is automatically discharged from GR?' is equal to 'No, the maximum length varies by facility' answer this question:***<br>What is the maximum rehabilitation length after which a post-COVID patient is automatically discharged from GR in the facilities participating in the EU-COGER study?	
1.17.2	***If 'Is there a nationally standardized maximum rehabilitation length after which a patient is automatically discharged from GR?' is equal to 'Yes' answer this question:***<br>What is the maximum rehabilitation length after which a post-COVID patient is automatically discharged from GR?	days

1.18	Is there any additional information that you would like to share with us?	

Thank you for filling in these questions
