# Supplementary material for: Post-COVID-19 recovery and geriatric rehabilitation care: a European inter-country comparative study
Source: Eur Geriatr Med. 2024 Aug 13;15(5):1489–501. doi: 10.1007/s41999-024-01030-w (PMC11614975; doi:10.1007/s41999-024-01030-w)
Supplement: Supplementary file 2 — Supplementary file2: Appendices I, II, IV, V, VI (DOCX 45 KB) [file 41999_2024_1030_MOESM2_ESM.docx]

**Appendix I. EU-COGER consortium list**

| Name healthcare organization | Country | Professional 1 | Professional 2 | Professional 3 | Professional 4 |
| --- | --- | --- | --- | --- | --- |
| Vseobecna fakultní nemocnice | Czech Republic | Eva Topinková | Lucie Bautzká | Helena Michaálková |  |
| Agaplesion Bethanien Hospital | Germany | Stefan Grund | Thomas Mross | Lotte Feesche |  |
| Robert-Bosch-Krankenhaus | Germany | Rebekka Leonhardt | Clemens Becker |  |  |
| Geriatrisches Zentrum Karlsruhe | Germany | Jan Gerhardus | Brigitte R. Metz |  |  |
| Geriatrische Rehabilitationsklinik Diakonissenkrankenhaus Mannheim | Germany | Diana Franke-Chowdhury |  |  |  |
| University of Limerick Hospital Group (ULHG) | Ireland | Rose Galvin | Aoife McCarthy |  |  |
| Beaumont Hospital | Ireland | Frances Dockery | Kara McLoughlin |  |  |
| Fliman geriatric rehabilitation center | Israel | Bahaa Francis |  |  |  |
| IRCCS Istituti Clinici Maugeri | Italy | Matteo Cesari | Annalisa Valentini |  |  |
| Karin Grech Hospital | Malta | Mark Vassallo | Maria Bonnici |  |  |
| Russian Clinical and Research Center of Gerontology | Russia | Olga Nikolaevna Tkacheva | Ksenia Eruslanova |  |  |
| Moscow Rehabilitation center | Russia | Luba Matchekhina |  |  |  |
| Parc Sanitari Pere Virgili | Spain | Laura Monica Perez Bazan |  |  |  |
| Hospital Universitari Sant Joan de Reus | Spain | Esther Roquer Fanlo |  |  |  |
| Hospital Universitari Parc de Salut Mar | Spain | Anna Renom Guiteras | Lizzeth Angela Canchucaja |  |  |
| Hospital Central de la Cruz Roja San José y Santa Adela | Spain | Beatriz Pallardo | Sergio Martínez Zujeros |  |  |
| Hospital San Joan de Deu Mallorca | Spain | Margarita Viñuela | Oriol Miralles Resina |  |  |
| Hospital Guadarrama | Spain | Gema Isabel Dominguez | Sarah Caro Bragado |  |  |
| Hospital de Barcelona | Spain | Nadia Stasi | Jennifer Garrillo Cepeda |  |  |
| Consorci Sanitari Alt’Pènedes i Garraf | Spain | Marta Arroyo-Huidobro | Ana Gonzalez |  |  |
| Leiden University Medical Center | the Netherlands | Wilco Achterberg | Monique Caljouw | Miriam Haaksma | Lisa van Tol |
| Omring | the Netherlands | Saskia Drijver |  |  |  |
| Zorgcirkel | the Netherlands | Paula Vonk |  |  |  |
| BrabantZorg | the Netherlands | Liesbeth Sikken | Irma Baars |  |  |
| Ijsselheem | the Netherlands | Nathalie Deden |  |  |  |
| Topaz Revitel | the Netherlands | Gerda Nijgh | Sylvia van der Drift |  |  |
| Tante Louise | the Netherlands | Heike de Wever | Els Calle |  |  |
| MUMC+\|Herstelzorg – Vitala+ | the Netherlands | Kaoutar Karramass | Josette Hendriks |  |  |
| Maastricht University | the Netherlands | Jos Schols | Irma Everink |  |  |
| Axion continu | the Netherlands | Lauren Ebbes |  |  |  |
| TriviumMeulenbeltZorg Almelo | the Netherlands | Anne Hartman | Hatice Koc |  |  |
| TriviumMeulenbeltZorg Hengelo | the Netherlands | Laura de Vries |  |  |  |
| Patyna | the Netherlands | Hylco Bouwstra |  |  |  |
| Careyn | the Netherlands | Laura Langendoen-Wigman |  |  |  |
| Sensire | the Netherlands | Berber Oldenbeuving | Sabine Noordam-Hemeltjen |  |  |
| Azora | the Netherlands | Liesbeth Lanting | Lulu Andela |  |  |
| Argos Zorggroep | the Netherlands | Mathilde Meerkerk |  |  |  |
| Meriant (Alliade) | the Netherlands | Lianne Willemstein | Krisztina Krasznai |  |  |
| Liemerije | the Netherlands | Janneke Wolting |  |  |  |
| Laurens Intermezzo Zuid | the Netherlands | Janette Tazmi |  |  |  |
| de Wever | the Netherlands | Eveline Keustermans |  |  |  |
| Icare – De Boshof | the Netherlands | Janetta de Vries | Sanne van Weers |  |  |
| SVRZ ‘t Gasthuis | the Netherlands | Lenni Boogaard |  |  |  |
| De Betuwe, Zorgcentrum Beatrix | the Netherlands | Simone Been |  |  |  |
| Archipel Zorggroep | the Netherlands | Danielle Termeer |  |  |  |
| Florence | the Netherlands | Patricia te Pas | Eva Lodewijks |  |  |
| Pieter van Foreest, locatie Bieslandhof | the Netherlands | Jeroen van den Berg |  |  |  |
| Reactiveringscentrum Klimop | the Netherlands | Sandra Prent | Marloes Boontje |  |  |
| Zorgspectrum Nieuwegein | the Netherlands | Joël Harms | Jeffrey Bakker |  |  |
| Zorggroep Maas en Waal | the Netherlands | Carolien de Croon |  |  |  |
| Attent | the Netherlands | Christa van Schieveen |  |  |  |
| Vivium Flevoburen (Zorggroep Almere) | the Netherlands | Ewout Smit |  |  |  |
| Kennemerhart Schoterhof | the Netherlands | Patricia van Berlo |  |  |  |
| Van Neynsel | the Netherlands | Dionne Ruchtie |  |  |  |
| Sheffield teaching Hospitals | UK | Jane Manson |  |  |  |
| Frimley Health NHS Foundation Trust | UK | Maria Espasandin | Lucy Abbott |  |  |
| Harrogate District Hospital | UK | Sarah Chadwick | Rebecca Watts |  |  |
| Imperial College Healthcare NHS Trust | UK | Melani Dani | Jackie McNicholas |  |  |
| University Hospitals of Derby and Burton | UK | Adam Gordon |  |  |  |
| Calderdale & Huddersfield | UK | Vincent Chau |  |  |  |
| Derbyshire Community Health Services | UK | Andy Cole |  |  |  |

**Appendix II: Missing data**

| **Table I.** Missing data in characteristics of post-COVID-19 patients in geriatric rehabilitation (GR) | | | | | | | | | | | |
| --- | --- | --- | --- | --- | --- | --- | --- | --- | --- | --- | --- |
|  | **ALL** | **CZ** | **DE** | **IE** | **IL** | **IT** | **MT** | **NL** | **RU** | **ES** | **UK** |
| **Number of participants per country, n (%)** | **723 (100)** | **53 (7.3)** | **50 (6.9)** | **50 (6.9)** | **32 (4.4)** | **30 (4.1)** | **17 (2.4)** | **293 (40.6)** | **50 (6.9)** | **96 (13.3)** | **52 (7.2)** |
|  |  |  |  |  |  |  |  |  |  |  |  |
| **Patients with missing data on…, n(%)** |  |  |  |  |  |  |  |  |  |  |  |
| Age |  |  |  |  |  |  |  |  |  |  |  |
| Sex, male, n (%) |  |  |  |  |  |  |  |  |  |  |  |
| Number of comorbidities, FCI* | 89 (12.3) |  | 11 (22.0) | 3 (6.0) |  | 1 (3.3) | 1 (5.9) | 58 (19.8) | 2 (4.0) | 5 (5.21) | 8 (15.4) |
| Frailty at GR admission, CFS** | 230 (31.8) |  | 1 (2.0) | 19 (28.0) | 19 (59.0) | 1 (3.3) |  | 168 (57.3) | 10 (20.0) |  | 12 (23.1) |
| Lived at home premorbid | 3 (0.4) |  |  |  |  |  |  |  | 1 (2.0) |  | 2 (3.8) |
| Hospital stay before GR admission, n (%) | 1 (0.1) |  |  |  |  |  |  | 1 (0.3) |  |  |  |
| Hospital length of stay prior to GR, n (%) | 78 (10.8) | 8 (15.1) | 1 (2.0) | 11 (22.0) |  |  | 1 (5.9) | 30 (10.2) | 22 (44.0) | 3 (3.13) | 2 (3.8) |
| ICU stay prior to GR, n (%) | 12 (1.7) |  |  |  |  | 1 (3.3) |  | 11 (3.8) |  |  |  |
|  |  |  |  |  |  |  |  |  |  |  |  |
| **Treatment components**, n (%) | 53 (7.3) |  | 4 (8.0) | 3 (6.0) |  |  |  | 44 (15.0) |  |  | 2 (3.8) |
|  |  |  |  |  |  |  |  |  |  |  |  |
| **Duration GR**, weeks, n (%) | 32 (4.4) |  | 2 (4.0) | 1 (2.0) | 3 (9.4) |  |  | 23 (7.8) |  | 1 (1.04) | 2 (3.8) |
|  |  |  |  |  |  |  |  |  |  |  |  |
| **Daily functioning**, Barthel Index, n (%) |  |  |  |  |  |  |  |  |  |  |  |
| at GR admission | 9 (1.2) |  |  |  |  |  |  | 8 (2.7) |  |  | 1 (1.9) |
| at GR discharge | 68 (9.4) |  | 4 (8.0) | 6 (12.0) |  |  |  | 56 (19.1) |  | 1 (1.04) | 1 (1.9) |
| **Quality of Life**, EQ-5D-5L, n (%) |  |  |  |  |  |  |  |  |  |  |  |
| at GR admission | 252 (34.9) |  |  | 11 (22.0) | 30 (100.0) |  |  | 149 (50.9) |  | 11 (0.11) | 48 (92.3) |
| at GR discharge | 310 (42.9) |  | 4 (8.0) | 12 (24.0) | 30 (100.0) | 1 (3.3) |  | 198 (67.6) |  | 14 (14.6) | 49 (94.2) |
|  |  |  |  |  |  |  |  |  |  |  |  |
| **Discharge destination**, n (%) | 20 (2.8) |  | 2 (4.0) | 2 (4.0) |  |  |  | 15 (5.12) |  | 1 (1.04) |  |
| CZ= the Czech Republic, DE=Germany, IE=Ireland, IL=Israel, IT=Italy, MT=Malta, NL=the Netherlands, RU=Russia, ES=Spain, UK= the United Kingdom; *FCI=Functional Comorbidity Index; **CFS= Clinical Frailty Scale | | | | | | | | | | | |

**Appendix IV:** Glossary geriatric rehabilitation (GR) care facilities

| Nursing home/long term care facility | An inpatient rehabilitation and medical treatment centre staffed with trained medical professionals, providing round-the-clock care. It is a **long term** residence. |
| --- | --- |
| Skilled nursing facility | An inpatient rehabilitation and medical treatment centre staffed with trained medical professionals, providing 24-hour nursing supervision. It is a **temporary** residence. |
| Acute care hospital ward | A ward of an acute care hospital (i.e. a hospital that provides inpatient medical care and other related services for surgery, acute medical conditions or injuries) dedicated specifically to (geriatric) rehabilitation, staffed with trained medical professionals, providing 24-hour nursing supervision. It is a **temporary** residence. |
| Specialised rehabilitation facility | An inpatient centre dedicated specifically to (geriatric) rehabilitation, staffed with trained medical professionals, providing 24-hour nursing supervision. It is a **temporary** residence. |
| Intermediate care facility | An inpatient rehabilitation and medical treatment centre staffed with trained medical professionals, which does **not** provide 24-hour nursing supervision. It is a **temporary** residence which generally caters to patients who are mobile and need less care compared to patients in skilled nursing facilities. |
| Ambulatory / outpatient treatment | Care that doesn’t involve admission to an inpatient hospital bed. The patient visits the caregiver. |
| Home-based treatment | Care that doesn’t involve admission to an inpatient hospital bed. The caregiver visits the patient at home for treatment. |

**Appendix V: Model estimates for recovery in daily functioning and quality of life**

**Table S1.** Linear mixed model for change in daily functioning (Barthel Index) over time (N=699)

|  | **NULL model** | **COUNTRY MODEL*** |
| --- | --- | --- |
| **Fixed effects** | **Mean (SE)** | **Mean (SE)** |
| Intercept (at admission) | 11.55 (0.16) |  |
| Country |  |  |
| *Reference:* United Kingdom |  | 7.58 (0.47) |
| the Czech Republic |  | 4.18 (0.65) |
| Germany |  | 4.87 (0.66) |
| Ireland |  | 4.20 (0.65) |
| Israel |  | 1.00 (0.73) |
| Italy |  | 6.60 (0.77) |
| Malta |  | 1.76 (0.92) |
| Russia |  | 6.09 (0.66) |
| Spain |  | 2.87 (0.58) |
| The Netherlands |  | 4.60 (0.52) |
|  |  |  |
| Linear weekly rate of change shortly before admission | -3.22 (0.09) | -3.25 (0.09) |
|  |  |  |
| Linear weekly rate of change after admission | 0.88 (0.04) |  |
| Country |  |  |
| *Reference:* United Kingdom |  | 0.56 (0.14) |
| the Czech Republic |  | 0.61 (0.22) |
| Germany |  | 0.95 (0.24) |
| Ireland |  | 0.24 (0.19) |
| Israel |  | -0.16 (0.20) |
| Italy |  | 0.22 (0.22) |
| Malta |  | 0.08 (0.21) |
| Russia |  | 1.35 (0.36) |
| Spain |  | 0.36 (0.16) |
| The Netherlands |  | 0.28 (0.15) |
| **Random effects** |  | Mean (SE) |
| Intercept variance | 8.94 (2.99) | 5.21 (2.28) |
| Slope variance after admission | 0.14 (0.37) | 0.09 (0.31) |
| Residual variance | 11.93 (3.45) | 11.77 (3.43) |

*adjusted for mean centred age and sex

**Table S2.** Linear mixed model for change in quality of life (EQ-5D-5L) over time (N=481)

|  | **NULL MODEL** | **COUNTRY MODEL*** |
| --- | --- | --- |
| **Fixed effects** | **Mean (SE)** | **Mean (SE)** |
| Intercept (at admission) | 0.56 (0.01) |  |
| Country |  |  |
| *Reference:* Ireland |  | 0.36 (0.04) |
| the Czech Republic |  | 0.17 (0.05) |
| Germany |  | 0.28 (0.06) |
| Italy |  | 0.31 (0.06) |
| Malta |  | 0.22 (0.07) |
| Russia |  | 0.50 (0.05) |
| Spain |  | 0.12 (0.05) |
| The Netherlands |  | 0.15 (0.04) |
|  |  |  |
| Linear weekly rate of change after admission | 0.03 (0.00) |  |
| Country |  |  |
| *Reference:* Ireland |  | 0.03 (0.01) |
| the Czech Republic |  | 0.02 (0.01) |
| Germany |  | 0.02 (0.01) |
| Italy |  | 0.01 (0.01) |
| Malta |  | -0.01 (0.01) |
| Russia |  | 0.01 (0.02) |
| Spain |  | 0.02 (0.01) |
| The Netherlands |  | 0.00 (0.01) |
|  |  |  |
| **Random effects** |  | **Mean (SE)** |
| Intercept variance | 0.06 (0.24) | 0.04 (0.19) |
| Residual variance | 0.04 (0.19) | 0.04 (0.19) |

*adjusted for mean centred age and sex

**Appendix VI. Multilevel model equations**

level 1: Y_ij_= a + b*x_ij_ + c*age + d*sex + f_i_ + ε_ij_

where:

Y_ij_ = Barthel Index (BI) or EQ-5D-5L score (QoL) for individual *i* at time *j*

a = intercept parameter (BI or QoL at GR admission)

b = slope parameter

x_ij_ = weeks since admission for individual *i* at time *j*

c = parameter estimate for age

d = parameter estimate for sex

f = parameter estimate for country of individual *i*

ε= residual error for individual *i* at time *j*

level 2: a = a_0_ + a_i_

where:

a_0_ = fixed intercept (BI or QoL at GR admission)

a_i_ = random intercept for individual *i*

b = b_0_ + b_i_

where:

b_0_ = fixed slope

a_i_ = random slope for individual *i* (only in BI model, as this parameter resulted in non-convergence for QoL)
